# Nanoprecipitation of polymeric nanoparticle micelles based on 2-methacryloyloxyethyl phosphorylcholine (MPC) with 2-(diisopropylamino)ethyl methacrylate (DPA), for intracellular delivery applications

**DOI:** 10.1007/s10856-015-5480-9

**Published:** 2015-03-14

**Authors:** Jonathan P. Salvage, Christopher Thom, Andrew L. Lewis, Gary J. Phillips, Andrew W. Lloyd

**Affiliations:** 1 School of Pharmacy and Biomolecular Sciences, University of Brighton, Huxley Building, Lewes Road, Brighton, BN2 4GJ UK; 2 Biocompatibles UK Ltd, a BTG International plc Group Company, Innovation Group, Lakeview, Riverside Way, Watchmoor Park, Camberley, GU15 3YL UK

## Abstract

Biodistribution of nanoparticle-based intracellular delivery systems is mediated primarily by particle size and physicochemical properties. As such, overcoming the rapid removal of these by the reticuloendothelial system remains a significant challenge. To date, a number of copolymer nanoparticle systems based on 2-methacryloyloxyethyl phosphorylcholine (MPC) with 2-(diisopropylamino)ethyl methacrylate (DPA), displaying biomimetic and pH responsive properties, have been published, however these have been predominately polymersome based, whilst micelle systems have remained relatively unexplored. This study utilised nanoprecipitation to investigate the effects of solvent and buffer choice upon micelle size and polydispersity, and found using methanol produced monodisperse micelles of circa 70 nm diameter, whilst ethanol produced polydisperse systems with nanoparticles of circa 128 nm diameter. The choice of aqueous buffer, dialysis of the systems, extended storage, and exposure to a wide temperature range (5–70 °C) had no significant effect on micelle size, and the systems were highly resistant to dilution, indicating excellent colloidal stability. Optimisation of the nanoprecipitation process, post precipitation, was investigated, and model drugs successfully loaded whilst maintaining system stability. Subsequent in vitro studies suggested that the micelles were of negligible cellular toxicity, and an apparent cellular uptake was observed via confocal laser scanning microscopy. This paper presents the first report of an optimised nanoprecipitation methodology for the formation of MPC–DPA nanoparticle micelles, and in doing so achieved monodisperse systems with the size and physicochemical characteristics seen as desirable for long circulating therapeutic delivery vehicles.

## Introduction

The research and development of systemically administrable nanoparticulate-based intracellular delivery systems (NIDS) is driven by the need to overcome a number of key challenges. The hurdles faced by NIDS include dilution induced micelle dissociation, resulting in dose dumping, and the more problematic rapid removal from the circulatory blood stream by the reticuloendothelial system (RES), mediated by particle size and physichochemical properties [1]. Whilst polymeric micelle systems have been shown to be able to withstand dilution to low concentrations, and thus offer the potential for systemic stability [2, 3], overcoming the RES remains a challenge. The main organs of the RES, the liver, spleen, and bone marrow, facilitate the removal of conventional NIDS within minutes of intravenous injection [4].

Two major factors are involved in this rapid removal, (i) opsonisation of the particle, and (ii) particle size. Opsonisation of NIDS occurs rapidly after injection [5] resulting in a surface layer of proteins on the NIDS which promote recognition and phagocytosis by the macrophages of the RES. Particle size of the NIDS can modulate both mechanical filtration and RES-based macrophage removal. Micron size range particles face capillary filtration in the lungs and spleen [6, 7], with particles of 1–2 µm having been shown to induce maximum phagocytosis [8]. Therefore a sub-micron particle size is considered desirable for a long circulating NIDS. However particles above 200 nm are subject to mechanical filtration by the spleen [9], whilst those below 50 nm may be lost through the fenestrae that occur throughout the capillary network of the liver, spleen, and bone marrow [10, 11]. Thus the range of particle sizes from 50 to 200 nm in diameter appears to offer the prospect of resistance to RES clearance. However, it has also been suggested that the use of protective surface coating technologies to extend nanoparticle circulatory times is less effective for particles with diameters greater than 150 nm [9], are more effective for particles within a narrower 60 to 100 nm size range [12]. When a NIDS possesses long circulating properties, tumour targeting can be achieved via the enhanced permeation and retention (EPR) effect, which will result in the passive accumulation of the NIDS at the tumour site via the leaky vasculature [13]. The long circulating properties are desirable as it has been hypothesised that a minimum of six hours are required for there to be effective EPR accumulation [14].

A number of successful biomedical materials have been successfully developed utilising phosphorylcholine (PC) [15] which possess biomimetic biocompatibility achieved by mimicking the surface of natural phospholipid membrane bilayers [16]. This could, in principle, reduce the protein adsorption and cell activation of NIDS containing PC, and therefore avoid macrophage-mediated RES removal.

Since the early reports of PC containing MPC–DPA nanoparticle systems [17, 18], there has been significant progress on the development of these in the form of polymeric vesicles, akin to liposomes, known as polymersomes [19, 20], whereas research into their further development as micelle-based NIDS has been more limited. Nevertheless, progress has been made with regard to demonstrating the continued potential for MPC–DPA based micelles to be effective NIDS [21, 22]. As such, this paper reports on the development of MPC_100_–DPA_100_ based micelle systems, which we have previously demonstrated possess some of the key attributes desirable in a NIDS: including drug analogue loading and release, pH responsiveness, dilution stability, and low cellular toxicity; but which were found to be below 50 nm in diameter, and of a polydisperse nature, having been formed by via a pH titration method [17]. Herein this paper reports the novel development and optimisation of a rapid and straightforward nanoprecipitation based method to achieve reproducible production of monodisperse MPC–DPA micelles. In doing so, this study investigated the effects of solvent and buffer choice, hydrophobic model drug loading, system temperature, and polymer concentration on the particle dynamics of the polymeric micelles formed, and is to the best of our knowledge the first report of this MPC_100_–DPA_100_ nanoprecipitated micelle system.

## Materials and methods

### MPC_100_–DPA_100_ diblock copolymer

The 100–100 diblock length copolymer, comprised of 2-methacryloyloxyethyl phosphorylcholine (MPC) with 2-(diisopropylamino)ethyl methacrylate (DPA), was supplied by Prof Steven Armes (University of Sheffield, UK), having been synthesised via atom transfer radical polymerisation (ATRP), and characterised with nuclear magnetic resonance (NMR) and gel permeation chromatography (GPC), as detailed previously [17, 18], where ^1^H NMR and GPC indicated the block copolymer was well defined with Mn and polydispersity values of 51,000 and 1.27 respectively.

### Preparation of polymeric micelle systems via nanoprecipitation

#### Solvent ratio, time, and dialysis effect

Solutions of the MPC_100_–DPA_100_ copolymer (40 mg ml^−1^) were prepared in methanol (MeOH) (Fisher, UK) and ethanol (EtOH) (Fisher, UK), and MeOH:EtOH volume combination ratios of 3:1, 1:1, and 1:3. Aliquots (100 µl) of these were added drop-wise, using a micropipette, to 9.9 ml of phosphate buffer saline (PBS) (Oxoid, UK), pH 7.4, stirring with a 1 cm magnetic flea (1,250 rpm, 2 min) and subsequently bath sonicated for 5 min. Final copolymer concentration was 0.4 mg ml^−1^. The systems were examined at time zero (t = 0) and at 6 weeks post micelle formation (t = 6), (room temperature storage), and also pre and post 1 week of dialysis, (8 kDa molecular weight cut-off) (BioDesign, USA), in PBS (pH 7.4) with daily PBS changes, using photon correlation spectroscopy (PCS).

#### Solvent, buffer, hydrophobic loading, dilution, and temperature effect

Solutions of the MPC_100_–DPA_100_ copolymer (40 mg ml^−1^) were prepared in MeOH and EtOH with and without a model hydrophobic compound Orange OT dye (OOT) (Sigma, UK), at a polymer to OOT mol ratio of 2:1. Aliquots (100 µl) of these were added drop-wise to 9.9 ml of PBS, pH 7.4, or MCIlvaines buffer (McB) (in-house), pH 7.4, stirring with a 1 cm magnetic flea (1,250 rpm, 2 min) and subsequently bath sonicated for 5 min. The systems were examined for dilution and temperature stability using PCS.

#### Stirring, sonication, and filtration effect

Solutions of the MPC_100_–DPA_100_ copolymer (40 mg ml^−1^) were prepared in MeOH, and aliquots (100 µl) of these were added drop-wise to 9.9 ml of PBS, pH 7.4, under the following preparation conditions: (A) no stirring, no sonication, no filtration, (B) no stirring, no sonication, 0.22 µm pore size syringe filtered (Millipore), (C) stirring (1,250 rpm, 2 min), sonication (5 min), no filtration, (D) stirring (1,250 rpm, 2 min), sonication (5 min), 0.22 µm pore size syringe filtered.

#### Loading ratio of fluorescent probe

Solutions of the MPC_100_–DPA_100_ copolymer (40 mg ml^−1^) were prepared in MeOH with the fluorescent probe Nile Red (NR) (Sigma, UK) at polymer to NR weight ratios of: (A) 10:0.1, (B) 10:0.5, (C) 10:0.75, (D) 10:1. Aliquots (100 µl) of these were added drop-wise to 9.9 ml of PBS, pH 7.4, with no stirring or sonication, and the resultant samples 0.22 µm pore size syringe filtered.

### Particle size characterisation

Particle size characterisation of the prepared micelle systems was undertaken using photon correlation spectroscopy (PCS), atomic force microscopy (AFM), and transmission electron microscopy (TEM).

#### Photon correlation spectroscopy

The PCS instrument employed in this study was a Malvern Zetasizer 3000HS, equipped with a 10 mW He–Ne laser operating at a wavelength of 633 nm, together with a high sensitivity avalanche photodiode detector (APD) at 90° collection angle. Sample temperature was maintained during PCS measurement by a Peltier thermal cuvette mounting stage. Each PCS size measurement had a duration of 600 s, consisting of 30 analyses by the Malvern PCS software, of fluctuations in the scattered light data collected by the APD, in order to determine the intensity based hydrodynamic diameter (D_h_) and system polydispersity (Pd). The D_h_ being calculated by the Stokes–Einstein equation (D_h_ = kT/3πηd), where D_h_ = hydrodynamic diameter, d = diffusion coefficient, k = Boltzmann’s constant, T = absolute temperature, and η = viscosity.

The solvent ratio, time, and dialysis effect systems (Sect. 2.2.1) were examined using PCS, at 25 °C, to determine the D_h_ and system Pd at time zero (t = 0) and at 6 weeks post micelle formation (t = 6), and also pre and post 1 week of dialysis (8 kDa cut-off) in PBS (pH 7.4) following daily PBS changes. Samples were filtered with 0.2 µm pore size syringe filters immediately prior to measurement to ensure sample quality.

The solvent, buffer, hydrophobic compound loading, dilution, and temperature effects on the systems (Sect. 2.2.2) were examined using PCS, at 25 °C, to determine D_h_ and system Pd in response to solvent and buffer variation, with and without OOT loaded in the systems. The dilution stability of the PBS systems was examined at 25 °C, by measuring signal intensity (KCps), D_h_ and Pd in response to halving dilutions of the micelle solutions made using pre-filtered (0.2 µm) PBS (pH 7.4). The diluted samples were filtered with 0.2 µm pore size syringe filters immediately prior to measurement, as before. The thermal stability of the PBS systems was examined by measuring the D_h_ and Pd across the temperatures range of 5–70 °C, at 5 °C intervals. Samples were equilibrated for 20 min after each 5 °C temperature change before PCS measurement commenced, to ensure only Brownian motion was measured. The stirring, sonication and filtration effects on the systems (Sect. 2.2.3) were examined using PCS, at 25 °C, to determine D_h_ and system Pd, without further sample filtration.

The effects of loading ratio of a fluorescent probe (Sect. 2.2.4) were examined using PCS, at 25 °C, to determine D_h_ and system Pd. Samples were filtered through a 0.2 µm pore size syringe filter immediately prior to measurement. Post instrument loading, the samples were allowed to equilibrate for 1 min per degree of temperature change, plus 5 min, from the room temperature before PCS measurement commenced at 25 °C.

#### Atomic force microscopy

MPC_100_–DPA_100_ dissolved in MeOH (40 mg ml^−1^) was added drop wise to pH adjusted deionised water (D.I.) under the same conditions used to prepare the PBS based system in Sect. 2.2.2. The micelle solution was then applied drop-wise to a mica wafer (in house) and air dried. The AFM imaging was carried out using a VEECO Nanoscope 111A atomic force microscope in Tapping Mode™, operating at a scan rate of 0.5 Hz, a scan size of 3 µm, and using Point probes type PPP-NCH-W tips (Nanosensors).

#### Transmission electron microscopy

The MPC_100_–DPA_100_ micelle solution, as described in Sect. 2.3.2, was mixed with 2 % phosphotungstic acid (pH 7.4) (Sigma, UK) at a 1:1 volume ratio as a negative stain, and then applied drop-wise to Formvar coated copper grids (Agar Scientific) and allowed to air dry. The prepared grids were imaged using a Phillips CM10 TEM at an accelerating voltage of 80 kV. Deionised water (pH adjusted) was employed for the AFM and TEM samples to minimise presence of salts upon drying.

### Cell study

#### In-vitro cell cytotoxicity study

The cytotoxicity assay used was based on published methods [23], and had been previously employed to successfully test MPC–DPA micelle systems [17]. MPC_100_–DPA_100_ (MeOH–PBS) (pH 7.4) based micelle systems were prepared at 0.4 mg ml^−1^, as per Sect. 2.2.2. Hamster lung V79 fibroblast cells (Japanese Cancer Research Resources Bank (JCRB), Japan) were maintained in Dulbecco’s Modified Essential Medium (DMEM) (PAA, UK) supplemented with 10 % foetal calf serum (FCS) (PAA, UK) and 1 % penicillin and streptomycin (P&S) (PAA Labs). 24 well plates (Nunc, UK) were seeded with 100 V79 cells per well in 500 µl of DMEM (10 % FCS, 1 % P&S) and incubated at 37 °C, in 5 % CO_2_ for 24 h to facilitate cell attachment. The 0.4 mg ml^−1^ micelle solutions were filter sterilised (0.22 µm) before preparing a series of 10-fold serial dilutions in DMEM (2.5 % FCS, 1 % P&S) to provide polymer concentrations of 200, 20, 2, 0.2 and 0.02 µg ml^−1^. After the 24 h cell attachment period, the supplemented DMEM was removed from the cells and replaced with 500 µl of the prepared dilutions of the polymer solutions, pre-warmed to 37 °C (four replicate wells per test concentration). Control samples of PBS and MeOH at the concentrations used for the polymer samples were also prepared and tested. A blank control plate containing cells in fresh DMEM supplemented with 2.5 % FCS and 1 % P&S was used to provide the 100 % survival figure. The plates were incubated at 37 °C in 5 % CO_2_ for 5 days to allow cell colonies to grow. The FCS concentration was reduced to 2.5 % from 10 % to attenuate cell growth. After 5 days the resultant colonies were fixed with gluteraldehyde (Sigma, UK) and stained with 10 % v/v Giemsa stain (Sigma, UK). The numbers of colonies in each well were counted and the percentage of colony reduction compared to the control wells was calculated for each dilution of each sample type.

#### Cellular uptake of dye loaded micelles

V79 cells were maintained in DMEM supplemented with 10 % FCS and 1 % P&S, and seeded into 24 well plates at 1,000 cells per well in 500 µl of DMEM (2.5 % FCS, 1 % P&S) and incubated at 37 °C, in 5 % CO_2_ for 48 h. MPC_100_–DPA_100_ (MeOH–PBS) micelle systems loaded with NR (10:0.5 w/w ratio) were prepared as per Sect. 2.2.4, substituting the PBS with DMEM (2.5 % FCS, 1 % P&S) in order to produce a final polymer concentration of 200 µg ml^−1^. The prepared polymer micelle solution, with NR, in DMEM (2.5 % FCS, 1 % P&S) was filter sterilised (0.22 µm), and used to replace the DMEM on the incubated cells. The cells were then incubated for a further 48 h at 37 °C, in 5 % CO_2_. At the 96 h time point, fresh DMEM was used to replace the test samples in the wells, having washed the wells twice with 1 ml sterile PBS beforehand. The cells were then observed using a Leica SP5 confocal laser scanning microscopy (CLSM) equipped with an argon laser, using an excitation wavelength of 514 nm, and an emission collection wavelength band of 550–617 nm.

## Results

### Solvent ratio, time, and dialysis effect

**Fig. 1:**
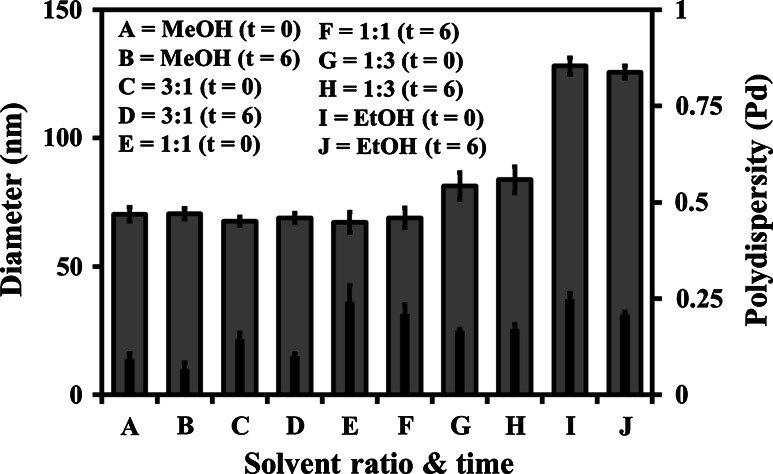
Particle diameter (*outer bar*) and polydispersity (*inner bar*), measured with PCS (25 °C), of MPC_100_–DPA_100_ micelles, formed via nanoprecipitation from solvent (MeOH, EtOH, and v/v combinations of), in PBS (pH 7.4), at time zero (t = 0) and 6 weeks post formation (t = 6) (Mean ± SD, n = 6)

**Fig. 2:**
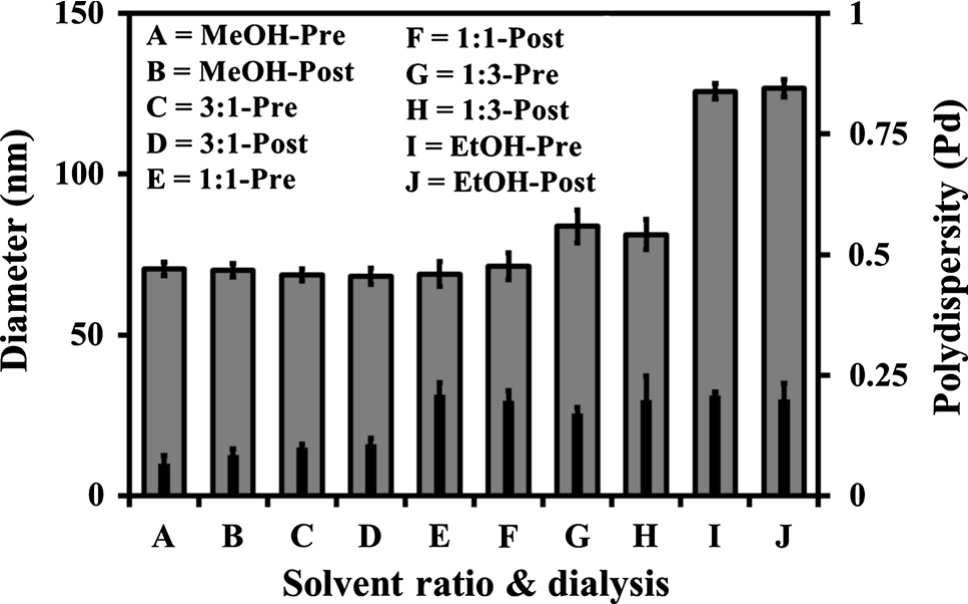
Particle diameter (*outer bar*) and polydispersity (*inner bar*), measured with PCS (25 °C), of MPC_100_–DPA_100_ micelles, formed via nanoprecipitation from solvent (MeOH, EtOH, and v/v combinations of), in PBS (pH 7.4), pre and post dialysis (Mean ± SD, n = 6)

A range of MeOH and EtOH solvent v/v ratio combinations were used to prepare micelle systems of MPC_100_–DPA_100_ in PBS. The PCS data (D_h_ and Pd) presented in Fig. 1 indicated that for systems prepared from 100 % MeOH, and MeOH:EtOH ratios of 3:1, and 1:1 the mean D_h_ was circa 70 nm. When the EtOH ratio was increased to 3:1 the D_h_ increased to a mean of circa 82 nm, and the 100 % EtOH system increased the mean D_h_ to circa 128 nm. The Pd of the 100 % MeOH derived system was below 0.1, indicating a monodisperse population, however as the EtOH ratio increased so did the Pd of the systems, going beyond 0.2 in some cases, indicating the development of polydisperse broader particle size range systems. Comparison of the t = 0 with 6 data presented in Fig. 1 indicated that the systems were stable over a 6 week storage period, with the D_h_ and Pd of the systems remaining relatively consistent for that period. The pre and post dialysis data displayed in Fig. 2 agreed with the data in Fig. 1, and indicated that dialysis of the systems had little discernable effect on their average D_h_ and Pd.

### Solvent, buffer, hydrophobic loading, dilution, and temperature effect

**Fig. 3:**
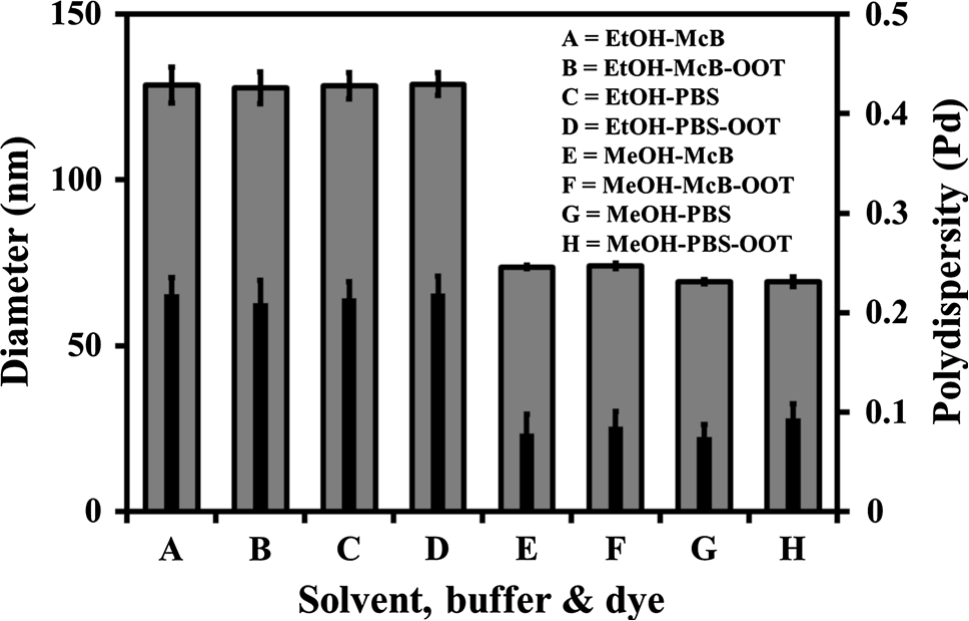
Particle diameter (*outer bar*) and polydispersity (*inner bar*), measured with PCS (25 °C), of MPC_100_–DPA_100_ micelles, formed via nanoprecipitation from solvent (MeOH or EtOH), in McB (pH 7.4) and PBS (pH 7.4), with and without OOT entrapped (Mean ± SD, n = 6)

MPC_100_–DPA_100_ micelle systems were prepared in either McB (pH 7.4) or PBS (pH 7.4) via nanoprecipitation using EtOH and MeOH, with and without OOT, as displayed in Fig. 3. The systems prepared using EtOH, both with and without OOT, and in PBS and McB, produced micelle systems with a mean D_h_ of circa 128 nm, and high Pd values exceeding 0.2, indicating the presence of polydisperse systems. The MeOH prepared systems, both with and without OOT, in PBS and McB produced micelle systems with mean D_h_ values of circa 70 and 74 nm, respectively, with low, monodisperse, Pd values below 0.1 for both systems. Thus the data in Fig. 3 indicated that the choice of buffer or loading with a hydrophobic compound (OOT) did not significantly alter the mean D_h_ and Pd of the either the EtOH or MeOH prepared systems, with only minor D_h_ changes observed when the buffer was switched for the MeOH systems.

**Fig. 4:**
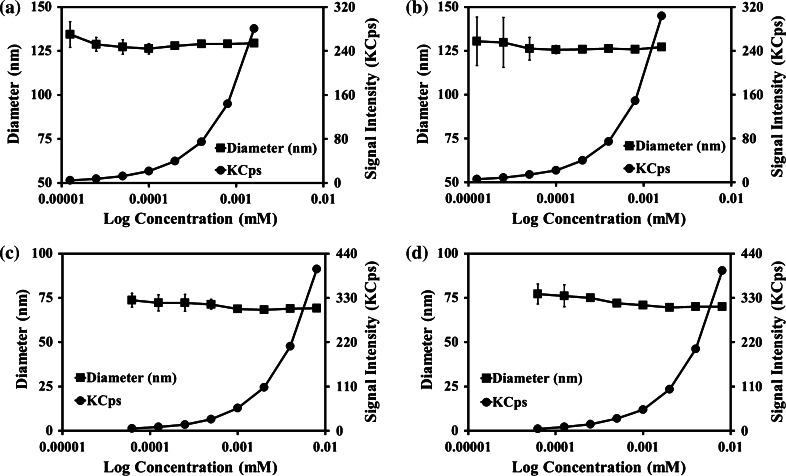
Particle diameter and signal intensity (KCps), in response to dilution, of MPC_100_–DPA_100_ micelles measured with PCS (25 °C), formed via nanoprecipitation from solvent into buffer. **a** Ethanol–PBS, **b** ethanol–PBS–OOT, **c** methanol–PBS, **d** methanol–PBS–OOT (Mean ± SD, n = 6)

The PBS based systems were further investigated, as displayed in Fig. 4(a–d) examining dilution stability of the micelles, with regard to the D_h_ and PCS signal intensity. The EtOH–PBS systems, without (a) and with (b) OOT loaded demonstrated good resistance to micelle dissociation as the polymer concentration was halved with each dilution point, resulting in a steady decrease in signal intensity. The MeOH–PBS systems, without (c) and with (d) OOT loaded also displayed resistance to dilution induced micelle dissociation, as was evident by measureable particles down to the limit of detection for the PCS instrument, circa 5 KCps.

For the EtOH–PBS systems, particles were detectable down to a concentration of 0.0125 µM (Fig. 4a, b), with an accompanying gradual reduction in the KCps signal intensity indicating that there were no sharp changes in mean particle size, which would have been seen upon a micelle to unimer shift, thus suggesting the persistent presence of micelles. The MeOH–PBS based systems were measurable down to 0.0625 µM, and also displayed a gradual reduction on signal intensity associated with particle stability.

**Fig. 5:**
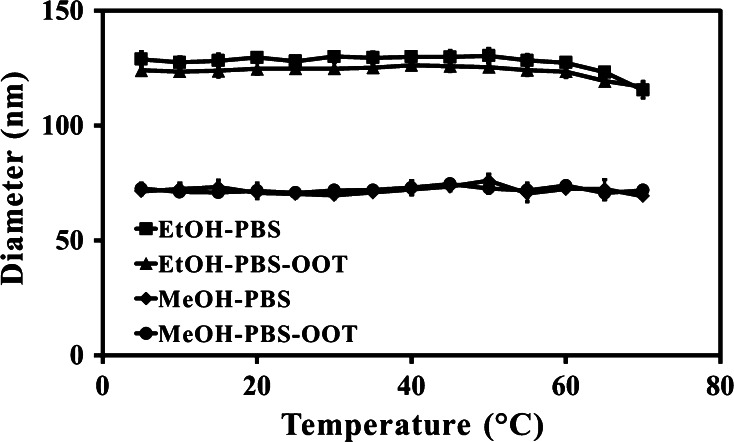
Particle diameter in response to temperature measured with PCS (5–70 °C), of MPC_100_–DPA_100_ micelles, formed via nanoprecipitation from solvent (MeOH or EtOH), in PBS (pH 7.4), with and without OOT entrapped (Mean ± SD, n = 6)

The systems were assessed for their temperature stability, with regard to D_h_, across the temperature range of 5–70 °C, as shown in Fig. 5. The EtOH–PBS systems, with and without OOT, remained stable throughout the temperature, with only a minor reduction in D_h_ at the highest temperature of 70 °C, with average D_h_ otherwise remaining circa 128 nm for the systems. The MeOH–PBS system, with and without OOT, also remained stable over the tested temperature range, with average D_h_ of circa 70 nm.

### Atomic force microscopy (AFM) and Transmission electron microscopy (TEM)

**Fig. 6:**
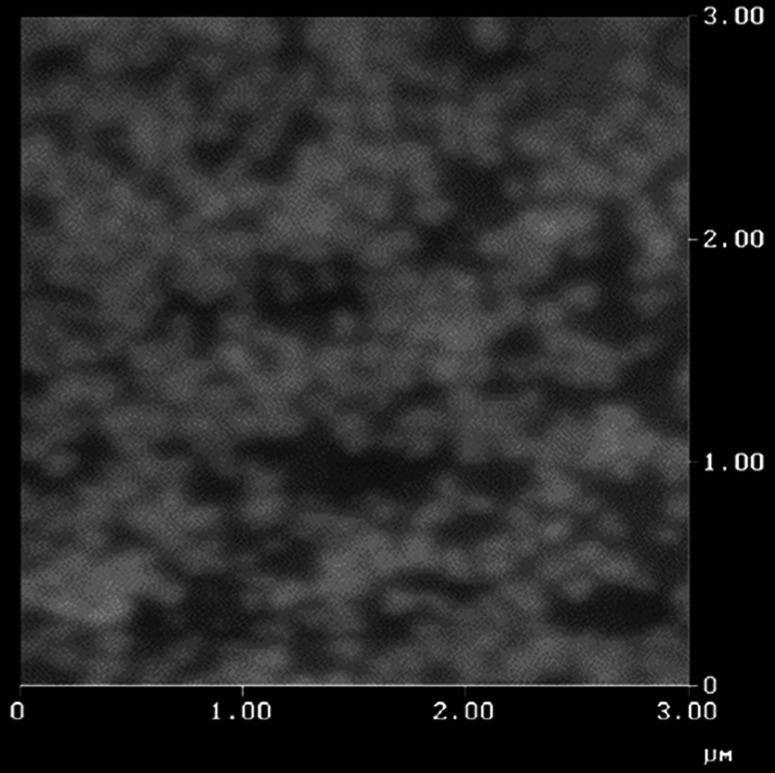
AFM Tapping Mode™ image of MPC_100_–DPA_100_ nanoparticle micelles displaying diameters in the order of 60–70 nm

**Fig. 7:**
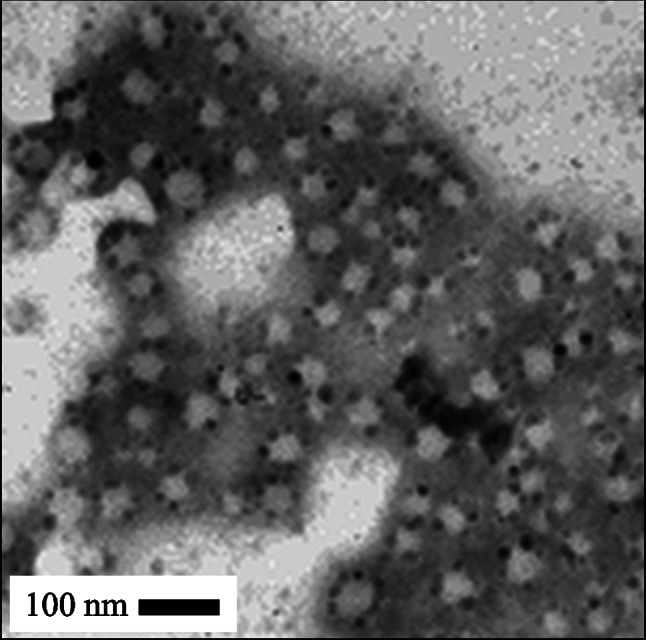
TEM image of MPC_100_–DPA_100_ nanoparticle micelles displaying micelle diameters in the order of 50–80 nm

AFM and TEM imaging was undertaken to further explore the size and morphology of the MPC_100_–DPA_100_ MeOH system. AFM imaging revealed a uniform population of nanoparticles with a spherical morphology, seen as light particles against a darker background in Fig. 6. TEM imaging also indicated the presence of spherical particles, seen as light against the dark background in Fig. 7. The particle size indicated by AFM and TEM was circa 50–80 nm.

### Stirring, sonication, and filtration effect

**Fig. 8:**
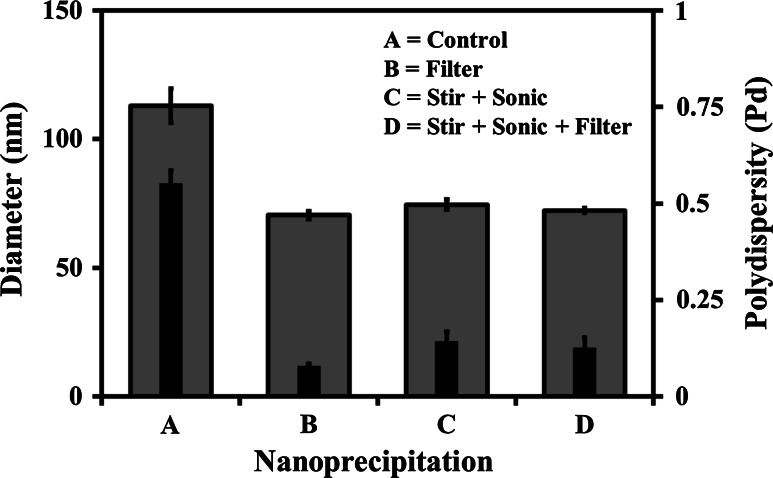
Particle diameter (*outer bar*) and polydispersity (*inner bar*), measured with PCS (25 °C), of MPC_100_–DPA_100_ micelles, formed via nanoprecipitation from MeOH in PBS (pH 7.4), illustrating the effect of filtration, stirring, and sonication on the D_h_ and Pd (Mean ± SD, n = 6)

To further optimise the preparation process the effects of stirring, sonication, and filtration upon D_h_ and Pd of the MeOH–PBS based system was examined. The results displayed in Fig. 8 show that the control samples (A), prepared using no stirring, sonication, or filtration during or after nanoprecipitation resulted in systems with average a D_h_ of circa 113 nm, and high mean Pd value of circa 0.6, suggesting the presence of a polydisperse system with larger, less well ordered, particle sizes. However, the samples prepared using filtration alone (B), stirring and sonication with no filtration (C), and stirring, sonication, and filtration combined (D), all resulted in systems with average D_h_ values circa 70 nm and low Pd values. The results indicated that it was only necessary to filter (0.22 µm) the system after nanoprecipitation in order to reproducibly produce monodisperse systems (Pd below 0.1), thus removing the requirement for the previous stirring and sonication steps.

### Loading ratio of fluorescent probe

**Fig. 9:**
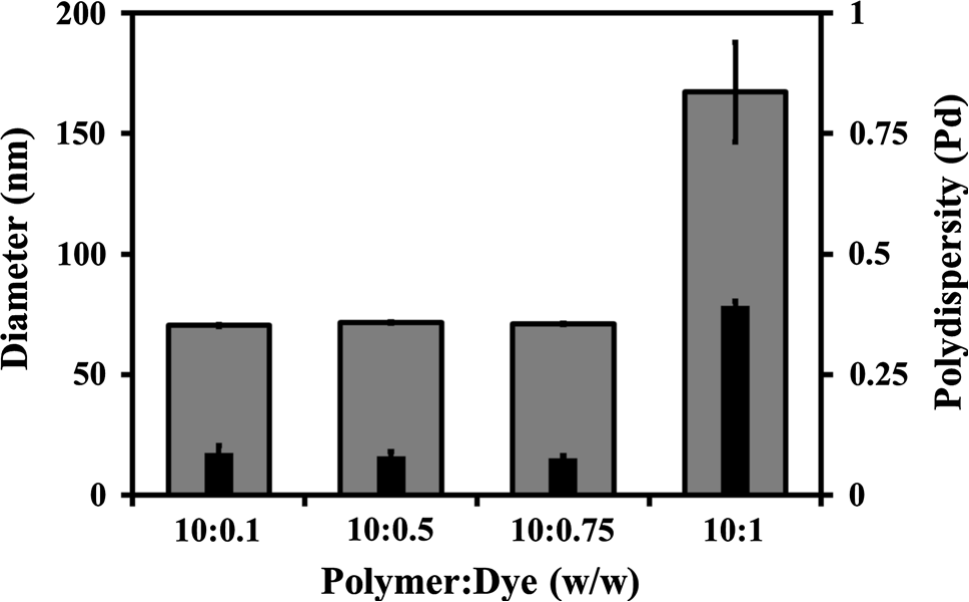
Particle diameter (*outer bar*) and polydispersity (*inner bar*), measured with PCS (25 °C), of MPC_100_–DPA_100_ micelles, loaded with Nile Red, formed via nanoprecipitation from MeOH in PBS (pH 7.4), illustrating the effect of alterations to the polymer:dye w/w loading ratio upon the D_h_ and Pd (Mean ± SD, n = 6)

In order to assess cellular uptake of the MPC_100_–DPA_100_ MeOH–PBS system, the fluorescent dye Nile Red (NR) was loaded into the system. Figure 9 illustrates the effect that alterations in the polymer:dye ratio had on the D_h_ and Pd of the system. It can be seen that at the w/w ratios of 10:0.1, 10:0.5, and 10:0.75 the system remained stable, with a D_h_ of circa 70 nm and Pd below 0.1, indicating monodispersity. At the ratio of 10:1 the D_h_ increased markedly to circa 167 nm, with a Pd of circa 0.4, suggesting the presence of a polydisperse system with larger, less well ordered, particle sizes.

### Cytotoxicity and cellular uptake

**Fig. 10:**
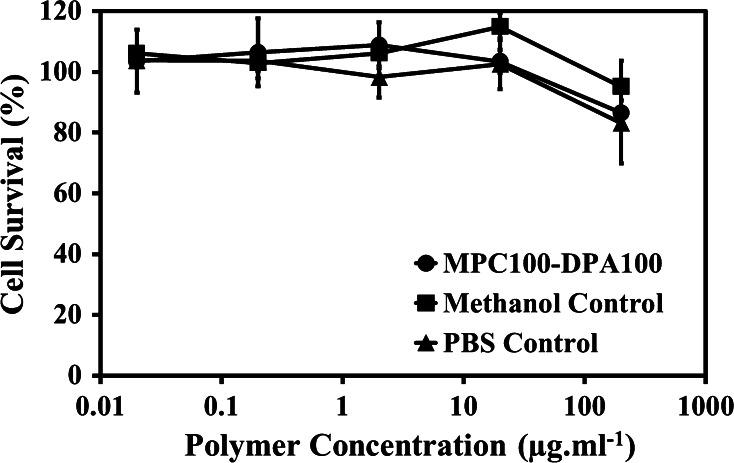
The in vitro cytotoxicity of the MPC_100_–DPA_100_ micelles (MeOH–PBS system) tested on V79 cells. The percent survival of test sample cells was derived from comparison to cell medium only control wells. Samples were incubated for 5 days at 37 °C in 5 % CO_2_ (Mean ± SD, n = 6)

The in vitro cytotoxicity of the MPC_100_–DPA_100_ micelles was investigated by comparing the cell colony formation numbers of the polymer micelle containing samples, with those of a polymer-free cell medium control plate. The data indicated that the micelles were essentially non-toxic at the tested concentrations, as seen in Fig. 10, and that the PBS and methanol controls also displayed similarly low levels of cellular toxicity at all but the highest concentration tested.

**Fig. 11:**
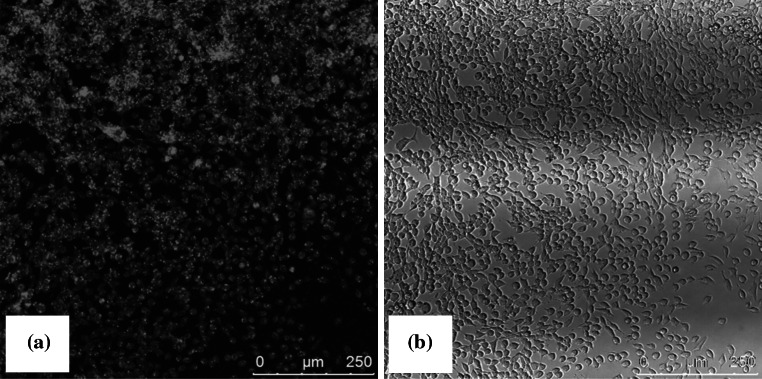
Confocal laser scanning microscopy image of V79 cells incubated with NR loaded MPC_100_–DPA_100_ micelles (MeOH–PBS system). Image **a** displays NR fluorescence, indicating apparent uptake of the NR loaded micelles, whilst image **b** provides brightfield evidence of healthy cell morphology

The cellular uptake of MPC_100_–DPA_100_ (MeOH–PBS) micelles loaded with NR florescent dye was investigated using CLSM and V79 cells. A seen in Fig. 11 an apparent uptake of the NR loaded micelles was observed, as evidenced by the NR fluorescence (a), whilst the brightfield image (b) of the same field of view indicated that the cells had maintained a healthy morphology.

## Discussion

Previous work has demonstrated that MPC–DPA copolymers molecularly dissolve in acidic solutions, form micelles when deprotonated in higher pH solutions, will dissociate when the pH is lowered again, and in doing so can be used to controllably load, deliver, and release hydrophobic compounds in vitro, whilst maintaining colloidal stability at physiological pH [17, 24]. Upon cellular internalisation, via endocytosis [25], the micelles are exposed to a low pH environment [26] which brings about micelle dissociation and drug release. It is therefore hypothesised that MPC–DPA micelles could be further developed and optimised to provide efficacious systemic delivery of clinically relevant therapeutic compounds, for example anticancer drugs, with recent in vivo studies continuing to support this concept [22]. The MPC component of the copolymer offers biomimetic and non-thrombogenic properties [15, 27, 28] which may provide the micelle corona with biocompatible performance in a similar manner to poly (ethylene glycol) (PEG) shielded Stealth liposomes [29], and thus in principle afford the MPC–DPA micelles a level of protection and defence against opsonisation induced RES clearance. Therefore the accurate and reproducible control of particle size, and polydispersity, is fundamental to effective biodistribution, and thus central to engineering long circulating MPC–DPA based NIDS, as particle size acutely influences RES clearance [1], particle accumulation at tumour sites via EPR [13, 14], and the rate and route of cellular internalisation via endocytosis [30]. Indeed, the poorly water soluble anti-cancer drugs tamoxifen and paclitaxel have been successfully loaded into other MPC–DPA based micelles [21, 24].

The principles of the nanoprecipitation method of micelle formation, also referred to as solvent displacement or solvent injection, have previously been employed to prepare a range of alternative nanoparticle systems [31–35] and was herein optimised for the polymer MPC_100_–DPA_100_. In this study nanoprecipitation proved to be an effective, rapid and straight forward micellisation technique, producing monodisperse micelle systems with mean diameters of circa 70 nm when using MeOH as the solvent and PBS as the non-solvent. This was a significant improvement over the previously employed pH titration method of MPC–DPA micelle formation which had produced smaller micelles with a higher level of polydispersity [17]. Interestingly, EtOH-based systems produced larger particles than the MeOH derived systems (Fig. 1), an effect which has also been reported for other nanoprecipitation based polymeric micelle systems exploring solvent selection [34, 35] and attributed to differing solubility of the polymers between solvents. PC based polymers have been reported to be soluble across the full range of methanol:water ratio mixes, whilst being less soluble in ethanol:water mixes, credited to the affinity of the PC headgroup to the alcohols relative to the water content, and the resultant co-nonsolvency effects [36, 37], which may account for the contrasting results observed in this work. Moreover, these findings were in good agreement with other work, that found the choice of solvent could influence particle size, and blends of solvents could be used to control particle size [34, 35, 38].

The maximum theoretical length of an MPC_100_–DPA_100_ unimer is 60 nm fully extended, based on a 0.15 nm carbon to carbon length, with each residue joined at a carbon bond and with one carbon (0.15 nm) between residues. However taking into account the 120° carbon bond angle, the distance between each residue of the polymer would be 0.26 nm, and therefore the unimer length would be 52 nm. In micelle form if the aggregated unimers were unfolded and had no hydrophobic domain overlap, the maximum theoretical micelle diameter would be 104 nm, herein the PCS measurements indicated the MeOH based particles were circa 70 nm, and EtOH system particles were circa 128 nm in diameter. Therefore whilst the MeOH system particles fit the size profile for micelles, the EtOH systems exceeded the maximum theoretical micelle diameter, and would thus appear to be more complex structures, possibly wormlike micelles or vesicles, further TEM imaging would help resolve this. Additionally any residual EtOH and MeOH in the buffer, post-injection, may have affected the solubility of the polymer, and thus lead to the difference in particle size observed, however the subsequent exhaustive dialysis (Fig. 2), utilised to remove residual solvent, indicated this was unlikely to be the case, as particle size remained unchanged post-dialysis. However, it is clear that choice of solvent can affect the size, and possibly morphology of the MPC–DPA particles, as reported for other systems [34, 35, 38, 39].

The EtOH and MeOH based systems were reproducible in both PBS and McB (Fig. 3), with only a slight reduction in particle diameter for MeOH in PBS versus the McB, but solution ionic strength is reported to influence micelle aggregation number, critical micelle concentration (CMC), and size [40, 41], however loading of the hydrophobic model compound OOT did not produce any discernable changes to the systems. Loading micelles with hydrophobic compounds can result in particle size changes [42–44] as has been seen in other MPC–DPA based micelle systems [24], however the previous micelle preparation method differed from that used for this study, which may account for apparent variance in particle size and stability. In contrast the MPC_100_–DPA_100_ systems were principally stable for both hydrophobic model compounds loaded, (Figs. 3, 9), until a 10:1 (w/w) polymer:dye ratio was reached (Fig. 9).

Polymeric micelle systems often possess very low CMC values and display good resistance to dissociation [2, 3]. The results of this study indicated that the MPC_100_–DPA_100_ micelles were highly resistant to dilution induced micelle dissociation, which is a major challenge for systemically administered self-assembling NIDS to overcome. The MPC_100_–DPA_100_ micelles were detectable, using PCS, down to polymer concentrations of 3.125 and 0.625 mg l^−1^ for MeOH and EtOH respectively in PBS, which were 10- and 100-fold improvements over the previous pH titration system CMC for the polymer [17]. The dilution stability values determined (Fig. 4) were also comparable to CMC values published for other MPC–DPA micelle systems, ranging from 4 to 25 mg l^−1^ [45, 46] as well as other copolymers containing either MPC or DPA ranging from 3.1 to 43 mg l^−1^ [47, 48]. Indeed it is possible that the micelle systems employed in this study would persist at lower polymer concentrations, as polymeric micelles have been found to have very low CMC values combined with high particle stability and slow particle dissociation [2, 3]; however the limit of detection for the PCS measurement was reached. Further study utilising pyrene solubilisation [36] would help elucidate this.

Temperature and time stability of candidate NIDS is of great importance if they are to be medically developed, as both thermal robustness and long term colloidal stability will influence the storage and handling requirements of the systems, and thus their ease of use in a full range of clinical settings. The MPC_100_–DPA_100_ micelle systems displayed good thermal stability, with no critical micelle temperature (CMT) or cloud points observed across a wide temperature range (Fig. 5), which was in contrast to other MPC–DPA micelle systems [49] which were reported to vary in particle diameter as temperature changed, possibly due to thermally induced micelle phase transitions [50], but again the micelle preparation method differed from that used for this study, as did the MPC–DPA block lengths, which may account for this variance. The thermal stability (Fig. 5), together with the stability over time observed (Fig. 1) indicate the MPC_100_–DPA_100_ systems have the potential to be adaptable to a range of storage and transport options.

In this current study the particle size for the MeOH based system indicated from the AFM imaging (Fig. 6) was in close agreement with the mean diameter (circa 70 nm) measured using PCS (Fig. 3), as was the TEM imaging result (Fig. 7). These TEM results were similar to reported diameters determined by TEM for other MPC–DPA micelles [24, 45, 46] but noting these systems were of different MPC–DPA block length ratios, and not prepared by using the nanoprecipitation method developed in this study. The negative staining technique used for the TEM imaging was an established method [3, 51, 52] and the resultant TEM image (Fig. 7) was comparable to other published negatively stained micelle TEM images, likewise the AFM image (Fig. 6) was also similar to other micelle system AFM images [53]. With regard to the wider particle diameter size range observed by TEM and AFM, compared to PCS, the micelles were fully hydrated and in an aqueous solution for PCS analysis, however during AFM and TEM sample preparation it is not unusual for micelles to spread, flatten, shrink, or contract during staining and drying, as they are not solid particles.

In order to optimise nanoprecipitation for the MPC–DPA micelles prepared from MeOH–PBS, the effect of stirring, sonication, and filtration upon the micelle solutions was examined (Fig. 8), all of which are commonly used methods published for micelle formation, and more so in the formation of polymersomes [20, 54, 55]. It was found in this case that there was no benefit observed from the stirring or sonication of the MPC_100_–DPA_100_ micelles, indeed it was found that sonication increased the polydispersity of the systems, possibly due to disruption of the micelles into a wider average diameter range, and that filtration was the only post-precipitation step required to achieve good monodispersity. The filtration step may have been acting as an extrusion process to induce the observed uniformity in the systems, as extrusion is one of a number of steps required to obtain uniform MPC–DPA and other polymersomes [54, 55]. In terms of further development, it has been reported that increasing the volume of solvent used for nanoprecipitation will increase the number or concentration of micelles in the solution, whilst increasing the polymer concentration will increase particle size [34, 35], which together with the solvent effects observed in this study, provides a large scope for further investigation.

The results from the in vitro cytotoxity assay (Fig. 10) indicated that the MPC_100_–DPA_100_ polymer was of very low cellular toxicity, which was in good agreement with other MPC–DPA polymers tested [17, 21, 56]. The small reduction in cell survival observed at 200 µg ml^−1^ polymer concentration was possibly due to the 50 % dilution of cell medium at the initial dilution step causing cell stress or reduced cell growth, rather than presence of the polymer, as the PBS control also displayed similar results. Similarly the MeOH used in sample preparation, and control volumes tested, also did not present any evidence of cellular toxicity over the PBS control. The V79 fibroblast cells may internalise nanoparticles via endocytosis [30], and an apparent uptake of the MPC_100_–DPA_100_ micelles was observed here using CLSM (Fig. 11a), with brightfield data (Fig. 11b) also displaying healthy cell morphology after exposure to the polymer, as further evidence of low cellular toxicity. Indeed a wide range of cell types have been exposed to MPC_25_–DPA_70_ polymersomes [54], none of which displayed any significant signs of toxicity, and thus the MPC_100_–DPA_100_ micelles should also be biocompatible. Whilst the existing MPC–DPA polymersome systems have a number of benefits, most notably the ability to deliver both hydrophobic and hydrophilic compounds [19, 20, 54, 56], the sample preparation required to attain the desired particle size and polydispersity is complex, whilst in contrast, these MPC_100_–DPA_100_ micelles offer the prospect of easily prepared reproducible monodisperse micelle systems, with the potential to deliver a wide range of poorly water soluble compounds.

Despite the small volume of MeOH used during the nanoprecipitation process, the potential toxicity of MeOH [57] cannot be ignored, and as such MeOH is often excluded from studies due to its inherent Class 3 toxicity rating [34]. However the results of this study are very promising in terms of controllable micelle system characteristics and properties, and this positive data warrants further investigation into methods to achieve and confirm removal of the MeOH. Moreover, given the cell assay data (Fig. 10) indicated that there was no residual toxicity from the MeOH used to prepare the micelles, it may be the case that MeOH dissipates from the system at ambient temperature. Additionally, given the boiling point of MeOH is approximately 65 °C, there was no evidence of micelle system shifts or alterations at 70 °C during PCS temperature scans (Fig. 5). The toxicity of the MeOH should also be taken into context of the application and the desired effect, for instance in the case of anti-cancer drug delivery, agents such as paclitaxel and doxorubicin are inherently toxic themselves, thus further investigation into understanding MeOH tolerance levels could be warranted, given low levels of unaccounted MeOH exposure occurs via environmental and dietary exposure. Indeed many pharmaceutical and medicinal compounds have serious side effects and wider health implications, but the benefit of treatment is perceived to outweigh these.

The MPC_100_–DPA_100_ diblock copolymer investigated in this study is essentially surfactant like, in that it is comprised of hydrophobic and hydrophilic domains, and in theory at a concentration above the CMC the unimeric polymer chains would to aggregate together to form micelles, with the hydrophobic blocks forming the core of the micelles [58]. The micellisation process is an example of the hydrophobic effect [59], where non-polar molecules or non-polar parts of molecules are spontaneously removed from water [60]. The primary driving force of this being the large energy required to form a cavity in the water to accommodate the hydrophobe, which is thermodynamically unfavourable and thus decreases the solubility of the hydrophobic molecules and favours micellisation [61]. The large energy is required because of the high cohesion of water molecules resulting from the high hydrogen bonding density [60]. The hydrophobic effect thus provides the driving force for the aggregation of the hydrophobic polymer domains, whilst electrostatic repulsion between the polar, water soluble, hydrophilic domains or head groups can control the shape and size the micelles [62]. Thus, in reality the shape of micelle based systems is controlled by the balance between these attractive hydrophobic and repulsive electrostatic forces [63]. The hydrophobic force will try to minimise the surface area to avoid water contact, whilst the repulsive force will try to maximise the surface area to keep the polar domains as far apart as possible [64]. This can ultimately lead to the spontaneous curvature of micelle formation, as the curvature allows a greater spacing between the polar domains than a planar bilayer [65]. In terms of drug loading, when a drug is mixed with micelles or surfactant molecules they may interact as a result of the hydrophobic effect and also electrostatic effects [66]. Drugs with no significant hydrophobic surface area may interact with the micelles or surfactants as a result of electrostatic effects arising from the charge associated with the drug and surfactant molecules. For drugs or hydrophobic compounds with a large hydrophobic surface area the hydrophobic effect will drive their interaction [66], although other chemical interactions may also occur, such a dipole interactions and hydrogen bonding depending on the chemical structure of the drug or surfactant [67]. The result of this hydrophobic effect is that poorly water soluble hydrophobic compounds, for example OOT and NR, will preferentially partition into the hydrophobic domain of the micelle core and undergo hydrophobic interactions [68].

Whilst the NR fluorescent probe employed in this study was hydrophobic, negligibly water soluble, and thus should preferentially partition into the core [68] of the MPC_100_–DPA_100_ micelles due to the hydrophobic effect [59–61], there is evidence from other MPC–DPA based micelle systems loaded with hydrophobic compounds, that a slow diffusion based release of the hydrophobic load at pH 7.4 can occur [24, 45]. However it should be noted that the studies [24, 45] were based on an excess volume dialysis model, rather than a fixed small volume and concentration system reflective of a tissue culture well. Given that micelles are generally considered to be thermodynamically stable above their CMC [45, 58], and noting that the polymer concentration utilised for the CLSM assay was 200 µg ml^−1^, with Fig. 4 indicating micelle stability at that concentration, it suggests that the MPC_100_–DPA_100_ micelles would persist at the concentration tested. Additionally, as the presence of hydrophobic compounds can enhance micelle stability [44], and the time data from this current study (Fig. 1) further suggested excellent colloidal stability, any micelle dissociation and NR release should therefore be minimal during the CLSM in vitro cell uptake assay.

However, the possibility remains that NR may have slowly diffused from the micelles during the assay incubation period, resulting in the cellular staining observed (Fig. 11). But, given the apparently rapid uptake of other MPC–DPA based systems [56], some of the NR loaded micelles may have undergone endocytosis into the cells and contributed to the fluorescence observed. As such, further study of the system with higher resolution microscopy would help elucidate this, as NR released in solution would be expected to primarily stain the lipid containing outer cell membrane, whereas micelle loaded NR should undergo cellular internalisation via endocytosis [21, 30, 56], and potentially enhance the level of fluorescent staining achieved [54]. Moreover, this could be further explored by preparing fluorescent dye labelled MPC–DPA [20], which would facilitate the investigation of both the fate of the micelle upon internalisation, and an additional second fluorescent dye loaded into micelle, and thus identify any co-localisation of the micelle and loaded dye.

Whilst the MeOH derived systems investigated here appeared to form surfactant-like spherical micelle structures, the larger particle diameter of the EtOH based systems suggests they may have formed more complex structures. It has been suggested for MPC–DPA polymers that when the DPA block length reaches approximately twice that of MPC block, then vesicle like polymersomes will form rather than micelles [49], indeed 140 nm diameter MPC_25_–DPA_120_ based vesicle-like particles have been reported [19] and a range of other polymersomes sizes greater than 100 nm diameter have been reported for MPC_25_–DPA_70_ [20, 54, 56] however in this instance, given the equal block length MPC_100_–DPA_100_ polymer, it would therefore be expected that micelles would be the predominate structure assembled.

The transition from spherical micelles to vesicles or polymersomes involves intermediate structures in the form of cylindrical or wormlike micelles [69] with solvent selectivity, block length and ratio [70], temperature, and the presence of salts and additives bringing about the transition of AB diblock copolymers from spherical micelles to a cylindrical or wormlike morphology [71, 72]. It is possible for rod or cylinder-like morphologies to further aggregate and form curved, bowl-shaped structures which can close over to form vesicles [73]. Therefore, given that the MPC–DPA polymer used for this study were also AB diblock copolymers it is possible that they may also have undergone transformation from spherical micelles to a cylinder or wormlike morphology and possibly through to vesicle formation, which may therefore account for the larger particle size seen for the EtOH based systems.

The principle concept for these MPC–DPA micelle systems, is that they are dynamic and controllable, and given the correct polymer, system conditions, and preparation parameters, have the potential to form a large number of organised structures [49] including spherical micelles, wormlike cylindrical micelles, and vesicles, all of which presents substantial scope for further investigation and research.

## Conclusions

In conclusion, progressing from work published previously on MPC–DPA micelles [17, 24] these results suggest that the MPC–DPA diblock copolymers continue to offer the potential for the development of long circulating stimuli responsive micelle NIDS. The reproducible monodisperse nature of the MPC_100_–DPA_100_ micelles, together with the demonstrated thermal and dilution stability, lends itself to the possibility that further designer micelles could be engineered to very specific diameters by alteration of the MPC and DPA block lengths. Indeed it has been suggested that the ratio between MPC and DPA is critical for the size and morphology of the particles formed [49], and as such developing accurate control of particle size and polydispersity is inherent to optimising control of NIDS bio-distribution.

This paper has described for the first time the development and optimisation of a novel preparation process, utilising nanoprecipitation, for monodisperse MPC–DPA micelle nanoparticle systems. These nanoparticles possess key biomimetic and pH responsive properties [17, 24] required for development of in vivo applications. OOT and NR were employed as model therapeutic compounds to test the systems, resulting in the successful formation of stable monodisperse micelles, with an apparent in vitro cellular delivery of these being demonstrated. These encouraging results highlight the continued potential for MPC–DPA systems to be utilised for a variety of intracellular delivery applications, for example anticancer, antimicrobial, and diagnostic imaging, harnessing the hydrophobic nature of many therapeutics to overcome their intrinsically low water solubility.
